# Antibiotic Resistance and Molecular Epidemiological Characteristics of *Streptococcus agalactiae* Isolated from Pregnant Women in Guangzhou, South China

**DOI:** 10.1155/2020/1368942

**Published:** 2020-04-30

**Authors:** Zhaomin Cheng, Pinghua Qu, Peifeng Ke, Xiaohan Yang, Qiang Zhou, Kai Lan, Min He, Nannan Cao, Sheng Qin, Xianzhang Huang

**Affiliations:** ^1^The Second Clinical College, Guangzhou University of Chinese Medicine, Guangzhou, China; ^2^Department of Laboratory Medicine, Guangdong Provincial Hospital of Chinese Medicine, Guangzhou, China; ^3^Department of Prenatal Diagnosis, Guangdong Provincial Maternity and Children's Hospital, Guangzhou, China

## Abstract

*Streptococcus agalactiae* colonization in pregnant women can cause postpartum intrauterine infections and life-threatening neonatal infections. To formulate strategies for the prevention and treatment of *S. agalactiae* infections, we performed a comprehensive analysis of antibiotic resistance and a molecular-based epidemiological investigation of *S. agalactiae* in this study. Seventy-two *S. agalactiae* strains, collected from pregnant women, were subjected to antibiotic susceptibility tests; then, the screened erythromycin and clindamycin nonsusceptible isolates were used for macrolides and clindamycin resistance genes detection, respectively. Detection of resistance genes, serotyping, and determination of virulence genes were performed by polymerase chain reaction. The clonal relationships among the colonized strains were evaluated by multilocus sequence typing. Matrix-assisted laser desorption/ionization time-of-flight mass spectrometry (MALDI-TOF MS) mass peak analysis was performed to discriminate the specific sequence types (STs). In our study, 69.4% and 47.2% of the strains were nonsusceptible to erythromycin and clindamycin, respectively; the multidrug resistance rate was 66.7%. All erythromycin nonsusceptible strains harbored resistance genes, whereas only 52.9% of the clindamycin nonsusceptible strains possessed the *linB* gene. Erythromycin resistance was mainly mediated by the *ermB* or *mefA/E* genes. Four serotypes were identified, and the most common serotype was serotype III (52.8%), followed by Ib (22.2%), Ia (18.0%), and II (4.2%). All the strains were divided into 18 STs that were assigned to nine clonal complexes. Most of the major STs were distributed into specific serotypes, including ST19/serotype III, ST17/serotype III, ST485/serotype Ia, ST862/serotype III, and ST651/serotype III. Analysis of virulence genes yielded seven clusters, of which *bca*-*cfb*-*scpB*-*lmb* (61.6%) was the predominant virulence gene cluster. Among all ST strains distributed in this region, only the ST17 strains had a mass peak at 7620 Da. The outcomes of this study are beneficial for the epidemiological comparison of colonized *S. agalactiae* in different regions and may be helpful for developing the strategies for the prevention of *S. agalactiae* infection in Guangzhou. Furthermore, our results show that MALDI-TOF MS can be used for the rapid identification of the ST17 strains.

## 1. Introduction

*Streptococcus agalactiae* is a microorganism that commonly colonizes in the gastrointestinal and genitourinary tracts of healthy women [1, 2]. *S. agalactiae* colonization in pregnant women is the main reason for intrauterine infection or transmission during parturition [3], which can cause serious neonatal infectious diseases, such as pneumonia, sepsis, and meningitis [4, 5]. Many countries have already issued guidelines for the prevention of maternal and neonatal *S. agalactiae* infections [6–8]. These guidelines generally recommend that pregnant women with *S. agalactiae* colonization should receive intrapartum antibiotic prophylaxis (IAP) to reduce the incidence of these infections [9, 10]. Currently, there are no official guidelines for *S. agalactiae* screening and prevention in China [5, 11]. However, in recent years, *S. agalactiae* has become an important pathogen in Chinese obstetrics and neonatology [12]. In mainland China, the *S. agalactiae* colonization rate of pregnant women ranged from 3.7 to 14.5%, and the incidence of invasive neonatal *S. agalactiae* diseases was 0.55–1.79 per 1000 live births, with a case fatality risk ranging from 6.5 to 7.1% [13]. The first-line antibiotic for IAP is penicillin [9, 10]; however, since some people are allergic to it, macrolides (e.g., erythromycin) and lincosamides (e.g., clindamycin) are used as the alternative antibiotics [14]. Unfortunately, the resistance of *S. agalactiae* to macrolides and lincosamides is increasing worldwide [4, 9, 11, 15, 16]. Previous studies have shown that the *ermB, ermTR*, and *mefA/E* genes are involved in the resistance to macrolides [17], and the *linB* gene has been linked to the resistance to lincosamides and moderate sensitivity to macrolides [18].

Capsular polysaccharide (CPS) is an important virulence factor of *S. agalactiae*, resulting in invasive infections [16, 19]. According to the different antigenicity of the CPS [20, 21] or by the multiplex polymerase chain reaction (PCR) assay [9, 22], *S. agalactiae* can be divided into 10 serotypes (Ia, Ib, and II to IX). Five of them (Ia, Ib, II, III, and V) are the predominant types of *S. agalactiae* that cause invasive infections [16, 23]. The understanding of the serotype distribution is pivotal in developing CPS-based polyvalent vaccines [16, 21]. In recent years, there have been a few reports on the serotype distribution of colonized *S. agalactiae* in China [11, 16, 24, 25]. Although there are some regional differences in the serotype distribution of *S. agalactiae* in these studies, similar common serotypes (III, Ia, V, and Ib) were observed. Several studies have shown that the virulence of *S. agalactiae* depends on not only the CPS, but also the surface-exposed bacterial proteins [26–29]. Several molecular typing methods have been applied to epidemiological studies, such as pulsed-field gel electrophoresis (PFGE), random amplification of polymorphic DNA, and multilocus sequence typing (MLST), of which MLST is a robust and convenient method that results in exchangeable data in different labs [30]. The combination of MLST and serotyping is a valuable method for *S. agalactiae* typing. For instance, the ST17 strain, belonging to serotype III, was found to be “hypervirulent” *S. agalactiae*, apparently associated with neonatal invasive diseases [31, 32]. Recently, whole-genome sequencing (WGS) has been used in genomic research of *S. agalactiae* [9, 33], such as accurate serotyping [34], in silico virulence investigation and antimicrobial susceptibility testing [33, 35], and speciation and evolutionary analysis [36]. However, at present, due to the high cost of WGS, it is not suitable for large sample analysis [34].

In recent years, matrix-assisted laser desorption/ionization time-of-flight mass spectrometry (MALDI-TOF MS), described as a “revolution in clinical microbiology” technology [32], has been widely used for bacterial identification based on protein fingerprints [37, 38], due to its accuracy, speed, and high throughput. This technology can also find characteristic proteomic biomarkers in some phylogenetic lineages of species and subspecies [38, 39]. For example, it was reported that “hypervirulent” *S. agalactiae* ST17 and emerging ST-1 clones could be rapid detected by MALDI-TOF MS [39].

The purpose of this study was to investigate the antibiotic resistance and resistance genes, serotype distribution, virulence, and genotyping of *S. agalactiae*, isolated from pregnant women in Guangzhou and establish rapid screening methods for some STs, which may be beneficial for the prevention and control of *S. agalactiae* infection.

## 2. Materials and Methods

### 2.1. Strains Collection and Identification

72 nonrepeating *S. agalactiae* strains were collected from pregnant women (35–37 weeks of pregnancy) in Guangdong Provincial Maternity and Children's Hospital, which is one of the major women's medical centers in Guangzhou, between January 2017 and December 2017. 1013 pregnant women were screened for colonized *S. agalactiae* during that time, and the carriage rate was 7.1% (72/1013).

The vaginal-rectal swabs collected from pregnant women were inoculated in Todd–Hewitt broth (bioMérieux, France), and the broth was incubated at 37°C in a 5% CO_2_ environment for 24 h. Afterwards, the samples were subcultured on Columbia agar, containing 5% sheep blood (bioMérieux, France), and the agar was incubated in the same conditions for 18–24 h. The suspected isolates were initially identified according to the following points: *β*-hemolysis (or nonhemolysis), colony morphology, and CAMP test [6]; the presumed *S. agalactiae* isolates were then confirmed by MALDI-TOF MS (VITEK®MS, bioMérieux, France).

### 2.2. DNA Extraction

The pure *S. agalactiae* cultures were harvested on Columbia agar, containing 5% sheep blood. The genomic DNA was extracted from each isolate using the MiniBEST Bacteria Genomic DNA Extraction Kit (TaKaRa, China) according to the manufacturer's instructions.

### 2.3. Serotyping

Serotyping by the multiplex PCR assay, as previously reported by Imperi et al. [22], was carried out for each isolate of *S. agalactiae* in this study. Those strains, which could not be classified into any serotype, were designated as nontypeable (NT).

### 2.4. MLST

Seven housekeeping genes (*adhP*, *pheS*, *atr*, *glnA*, *sdhA*, *glcK*, and *tkt*) were amplified to implement MLST, as described previously [26]. The obtained products were purified and sequenced using sanger sequencing by the Sangon Biotech (Shanghai) Co., Ltd. The allele numbers and STs were obtained using online data (http://pubmlst.org/sagalactiae/). The phylogenetic tree based on the concatenated sequences of the seven loci was constructed by the MEGA6 software using the UPGMA method.

The relationships among different STs were estimated using eBURST version 3.1 (http://eburst.mlst.net/v3/). Note that only those shared six identical alleles were defined as a clonal complex (CC) [40, 41]. Additionally, to understand the location of the STs for this study, the eBURST diagram of the *S. agalactiae* population was constructed using all STs found around the world, which are reported in the international database (http://pubmlst.org/sagalactiae/) (see Table S1 in the Supplementary Materials) as of August 2018.

### 2.5. Antibiotic Susceptibility Tests

All strains were assessed for susceptibility to penicillin (10 units), ampicillin (10 *μ*g), erythromycin (15 *μ*g), clindamycin (2 *μ*g), tetracycline (30 *μ*g), levofloxacin (5 *μ*g), vancomycin (30 *μ*g), linezolid (30 *μ*g), and chloramphenicol (30 *μ*g) (Oxoid, UK) by disk diffusion method, according to the recommendation of Clinical and Laboratory Standards Institute (CLSI) 2017 guidelines (http://www.clsi.org). The D-zone test was carried out for the strains nonsusceptible to erythromycin but susceptible to clindamycin. *Streptococcus pneumoniae* ATCC 49619 was used as a quality control strain to ensure the credibility of the results. Multidrug resistance of *S. agalactiae* was defined as acquired nonsusceptibility to at least three classes of antibiotics as previously described [42, 43].

### 2.6. Determination of Macrolides and Lincosamides Resistance Genes

The macrolides resistance genes *ermB*, *ermTR*, and *mefA/E* were tested by PCR in all erythromycin nonsusceptible isolates, while the lincosamides resistance gene *linB* was detected in each clindamycin-resistant strain. The primers that were used and the conditions in which the reactions were performed were as described previously [17, 44, 45].

### 2.7. Detection of Virulence Genes

Five major virulence genes that encode the surface-proteins, including toxins CAMP factor (*cfb*), *α*- (*bca*) and *β*-subunits (*bac*) of C protein, C5a peptidase (*scpB*), and laminin-binding protein (*lmb*), were analyzed using PCR. The specific experimental procedures used in the present study have been published earlier [29].

### 2.8. Characteristic Mass Peak Analysis

MALDI-TOF MS characteristic mass peak analysis was performed as briefly described below. First, single colonies from the overnight-cultured isolates were picked and coated duplicate on a target plate (VITEK®MSDS, bioMérieux, France). *α*-Cyano-4-hydroxycinnamic acid (CHCA; bioMérieux, France) (1 *μ*L) was added to the tested samples, and the target plate was left to dry. The RUO Mode (VITEK®MS RUO, Shimadzu, France) was then calibrated with *Escherichia coli* ATCC 8739 strain, and the mass spectrometry data of each strain were collected in a positive linear mode within a mass range from 3,000 Da to 20,000 Da [46]. The data were imported to SARAMIS premium software (bioMérieux, France), and the comparison of the mass peaks was carried out by Lanchpad software (Shimadzu Biotech, USA).

### 2.9. Statistical and Data Analyses

Fisher's exact test was used to evaluate the differences in antibiotic sensitivity and distribution of resistance genes among different serotypes. The relationships between CCs, serotypes, and related virulence gene profiles were analyzed by a correspondence analysis. All statistical analyses were performed with SPSS software version 22.0. A *P*-value <0.05 was considered statistically significant.

### 2.10. Ethical Statement

This study protocol was in accordance with the Helsinki Declaration of 1964. Data collected in this study did not include the information about patients, which was exempted from the formal medical ethical approval by the ethics committee of the hospital where the research was carried out (ZM2016-280).

## 3. Results

### 3.1. Serotype Distribution

Four serotypes were detected in 72 *S. agalactiae* isolates, and serotype III was the most frequently identified serotype, accounting for 52.8% (38/72). The proportions of serotypes Ia, Ib, and II were 18.0% (13/72), 22.2% (16/72), and 4.2% (3/72), respectively. The other two *S. agalactiae* isolates were NT.

### 3.2. MLST

**Figure 1:**
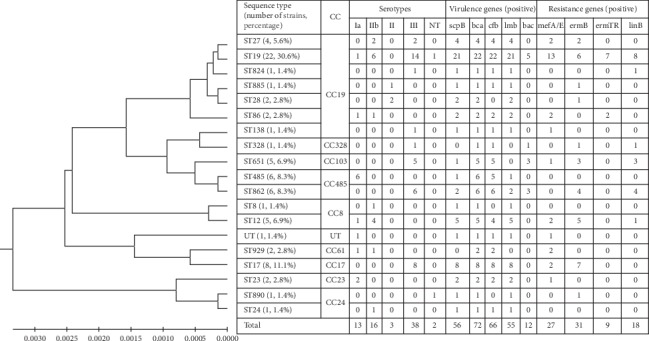
UPGMA dendrogram of the 72 *S. agalactiae* strains was constructed by MEGA6 software. This dendrogram shows the genetic diversity and molecular characteristics of different STs. Nonsusceptibility includes intermediation and resistance, and no strains were found intermediate to clindamycin in this study. ST, sequences type; CC, clonal complex; UT, untypable; NT, nontypeable; values express the number of strains.

Of the 72 *S. agalactiae* isolates in this study, 71 were classified into 18 unique STs. The allele combination of the remaining strain was *adhP* (1), *pheS* (1), *atr* (4), *glnA* (2), *sdhA* (1), *glcK* (3), and *tkt* (4). Since the database did not have an assigned ST for this allele combination, the strain could not be classified by MLST. As shown in Figure 1, the most frequently detected ST was ST19 (30.6%), followed by ST17 (11.1%), ST485 (8.3%), ST862 (8.3%), ST12 (6.9%), ST651 (6.9%), and ST27 (5.6%). The proportions of other STs (ST8, ST23, ST24, ST28, ST86, ST138, ST328, ST824, ST885, ST890, and ST929) were less than 3.0%, respectively.

**Figure 2:**
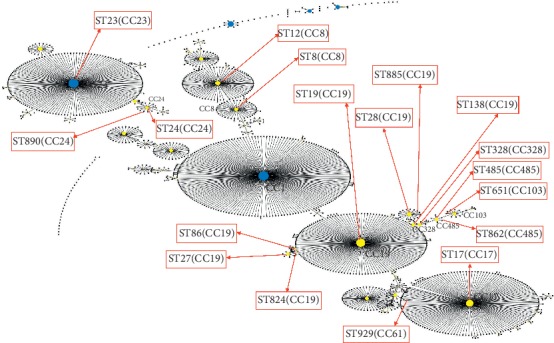
The eBURST diagram of *S. agalactiae*. This diagram is constructed using all STs found around the world as of August 2018. Number of STs = 1311. ST labels have been removed and the original diagram has been optimized by manual edited. Each dot represents a ST; blue and yellow dots represent founding and subgroup founding types, respectively. The red font represents the clonal complexes found in this study. Red boxes pointed by red arrows indicate the STs detected in this study and display which clonal complexes the STs belongs to.

Using the eBURST tool, a population snapshot was constructed to show the clusters of all known STs (number of STs = 1311; see Table S1 in the Supplementary Materials) in the entire *S. agalactiae* MLST database, and the relationship between the STs classified in this study and worldwide prevalent STs (see Figure 2) was elucidated. By eBURST analysis, 18 STs were assigned to 9 CCs (CC8, CC17, CC19, CC23, CC24, CC61, CC103, CC328, and CC485), with CC19 being the most prevalent (45.8%). The eBURST analysis displayed that CC17, CC61, CC103, CC328, and CC485 were close to CC19. Interestingly, CC19 and CC8 were close to CC1, while CC24 was close to CC23.

### 3.3. Antibiotic Susceptibility and Resistance Genes

All isolates were sensitive to penicillin, ampicillin, vancomycin, and linezolid. As shown in Table 1, the nonsusceptibility rates for erythromycin, clindamycin, tetracycline, levofloxacin, and chloramphenicol were 69.4%, 47.2%, 90.3%, 37.5%, and 31.9%, respectively. Notably, 66.7% (48/72) of all *S. agalactiae* isolates were multidrug resistant (MDR) (see Table S2 in the Supplementary Materials for antibiotic resistance combination). No strains were found to be intermediate sensitivity to clindamycin and levofloxacin in this study. In other words, the nonsusceptibility rates for clindamycin and levofloxacin were equal to the resistance rates. 19 isolates were nonsusceptible to erythromycin, but susceptible to clindamycin, and eight of them were positive for the D-zone test.

The macrolides resistance genes were determined for 50 isolates of erythromycin nonsusceptible *S. agalactiae*, and at least one macrolides resistance gene was detected in each strain. The major prevalent resistance genes were *ermB* (62.0%), followed by *mefA/E* (54.0%) and *ermTR* (18.0%) (see Table 1). Among the 34 clindamycin-resistant isolates, 18 strains (52.9%) carried the *linB* gene (see Table 1).

Overall, there were no significant differences in the antibiotic susceptibility, *ermB*, *mefA/E,* and *linB* resistance genes, except in the *ermTR* gene (*P*-value = 0.001), among the different serotype isolates (see Table 1).

### 3.4. Virulence Genes

As shown in Figure 1, the *bca* gene was detected in all the strains, and the *cfb* gene was frequently identified, accounting for 91.7% (66/72). The prevalence of the *scpB*, *lmb*, and *bac* virulence genes was 77.8% (56/72), 70.5% (55/72), and 16.7% (12/72), respectively. Intriguingly, the *lmb* gene was detected in almost all the *scpB* gene-positive strains. Based on the presence or absence of each virulence gene, all 72 strains were clustered into seven virulence gene profiles (see Table 2). The *bca*-*cfb*-*scpB*-*lmb* (61.6%), *bca*-*cfb* (12.5%), and *bca*-*cfb*-*bac* (9.7%) patterns were the main virulence gene profiles. Only five strains (6.9%) contained all five virulence genes.

### 3.5. Characteristic Mass Peak Analysis

MALDI-TOF MS analysis revealed the differences in the mass peaks among the different STs. Relative to the other STs, all ST17 strains (*n* = 8) were identified at the mass peak of 7620 Da (see Figure S1 in the Supplementary Materials). In other words, the mass peak of 7620 Da was the characteristic mass peak of the ST17 strains.

### 3.6. Relationships among the Various Molecular Characteristics of *S. agalactiae* Strains

According to the UPGMA dendrogram, the *S. agalactiae* strains isolated from pregnant women in Guangzhou were divided into two main branches (see Figure 2), namely, CC1 and CC23 (see Figure 1), which were further divided into different subbranches, representing the different STs (see Figure 2). Interestingly, some STs were closely related to specific serotypes (see Figure 2). For instance, the ST17, ST651, and ST862 strains were attributed to serotype III, while the ST485 and ST885 were strains classified as serotypes Ia and II, respectively.

**Figure 3:**
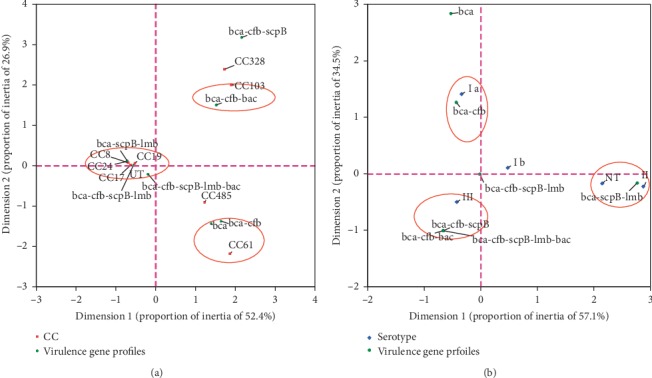
Correspondence analysis for the relationship between CC, serotypes, and virulence gene profiles. (a) CC and virulence gene profiles. (b) Serotypes and virulence gene profiles. CC, clonal complex; UT, untypable; NT, nontypeable.

The correspondence analysis indicated that there was a significant correspondence between the CCs and virulence gene profiles (*χ*^2^ = 106.52, *P*-value <0.001) and the serotypes and virulence gene profiles (*χ*^2^ = 51.61, *P*-value = 0.001) (see Figure 3). For example, the *bca*-*cfb*-*scpB*-*lmb* profile was distributed in CC17 and CC23, the *bca*-*cfa*-*bac* profile was distributed in CC103, the *bca*-*cfb* profile was distributed in serotype Ia, and the *bca*-*scpB*-*lmb* profile was distributed in serotype II.

Besides that, all erythromycin nonsusceptible isolates (*n* = 50, including ST27, ST19, ST885, ST28, ST86, ST138, ST651, ST862, ST12, ST929, ST17, ST23, and ST890) carried at least one macrolide resistance gene. Of these strains, 42.0% (21/50) carried the *ermB* gene, 24.0% (12/50) carried the *mefA/E* gene, 16.0% (8/50) carried both the *ermB* and *mefA/E* genes, 4.0% (2/50) carried both the *ermB* and *ermTR* genes, and 14.0% (7/50) carried both the *mefA/E* and *ermTR* genes (see Table 3). Erythromycin nonsusceptible strains were found in all the mainly prevalent STs (ST12, ST17, ST19, ST27, ST651, and ST862) (see Table 3), except ST485. Some of these mainly prevalent STs were obviously associated with specific macrolide resistance genes. All ST12, ST17, ST651, and ST862 erythromycin nonsusceptible isolates were found to carry the *ermB* gene (see Table 3 and Figure 1). However, only 31.3% (5/16) of the ST19 erythromycin nonsusceptibility isolates carried the *ermB* gene. The *mefA/E* gene was significantly related to erythromycin nonsusceptibility in ST19 than in the other mainly prevalent STs (ST12, ST17, ST27, ST651, and ST862) (*χ*^2^ = 11.99, *P*-value = 0.018), and 81.3% (13/16) of the ST19 erythromycin nonsusceptibility isolates carried the *mefA/E* gene (see Table 3 and Figure 1). Of the mainly prevalent STs, only some ST19 erythromycin nonsusceptibility isolates were found to carry the *ermTR* gene (see Table 3 and Figure 1). As mentioned earlier, only 52.9% (18/34) of all clindamycin-resistant isolates were found to carry the *linB* gene. Intriguingly, the *linB* gene was mainly found in ST19 (88.9%, 8/9), ST651 (100%, 3/3), ST862 (100%, 4/4), ST824 (100%, 1/1), and ST328 (100%, 1/1) clindamycin-resistant isolates. However, no *linB* gene was found in ST17, ST890, ST885, and ST28 clindamycin-resistant isolates.

## 4. Discussion

Maternal *S. agalactiae* colonization can lead to postpartum intrauterine infections and invasive neonatal diseases [3–5]. A systematic review and meta-analysis revealed that the carriage rate of *S. agalactiae* in pregnant women was 10% (95% CI 8, 12), with 7% in Asia and 19% in non-Asian countries [24]. In this study, the carriage rate of *S. agalactiae* in pregnant women in Guangzhou was found to be 7.1%. Although the carriage rate was not obviously different from the average rate in Asia, it is necessary to investigate the prevalence of *S agalactiae* in different regions of China, considering the huge population of China and limited research data of colonized *S. agalactiae*.

Previous reports have shown differences in the phenotypic and genotypic characteristics of colonized *S. agalactiae* from different geographical regions [16, 28, 47]. Accordingly, determining the population structure of colonizing isolates (e.g., genetic diversity, virulence factors, and antibiotic resistance) is the key to understand *S. agalactiae* disease in a region [9]. The present study is the first comprehensive investigation of the molecular epidemiological characteristics of colonized isolates in pregnant women in Guangzhou, South China.

Serotyping of *S. agalactiae* is crucial in determining the pathogenicity of the isolates [48]. In this study, four serotypes (Ia, Ib, II, and III) were identified by molecular serotyping. Overall, the three primary serotypes among the colonized isolates in Guangzhou were III, Ib, and Ia (93.0%), which were also reported as predominant serotypes in other Chinese cities, such as Dongguan (85.7%) [11] and Beijing (66.1%) [16]. However, the predominant serotypes in many other areas in other reports, including serotypes Ia, II, and Ib in Brazil (65.4%) [49] and serotypes III, V, and Ia in Toronto (67.0%) [9] and Shanghai (79.6%) [21], are different from those in our study. It has been reported that the colonization of serotype III isolates in pregnant women is a high risk factor for neonatal infections [12, 21]. Notably, the proportion of the most common serotype (serotype III) in our region (52.8%) was similar to that reported in adjacent Dongguan (54.9%) [11], but apparently higher than that reported in Shanghai (East China, accounting for 35.9%) [21] and Beijing (North China, accounting for 32.1%) [16]. These data suggest that the prevalence of serotype III isolates in South China may be higher than that in other parts of China. The CPS of *S. agalactiae* is not only a basis for serotyping, but also an important target for vaccine research. In the development of conjugated multivalent vaccines, only five serotypes (Ia, Ib, II, III, and V) were covered [50, 51]. Fortunately, all isolates in this study, except two NT strains, were hypothetically covered by a vaccine combination containing polysaccharide conjugates of the five serotypes. NT strains have also been reported in previous studies [2, 21, 52] and may be related to mutations in the CPS locus [9, 53].

Molecular epidemiological studies using MLST reveal the characteristics of the distribution of colonized *S. agalactiae* lineages. In this study, 18 distinct STs were identified, and these were then grouped into nine CCs. The colonized isolates from Guangzhou show relatively higher genetic diversity. Nonetheless, the three prevalent CCs, that is, CC19, CC485, and CC17, accounted for 73.6%. CC19 is the most common CC among asymptomatic pregnant women in our region, a finding similar to the global trend [2, 14, 47]. Interestingly, MLST analysis revealed that some bovine dominant subtypes had begun to spread in humans. ST485 was previously thought to be undetectable in humans, but recently, it has dramatically increased in pregnant women in China (2.5%–14.13%) [16, 33, 54]. In addition, some studies have shown that ST485 is highly pathogenic and may cause threatened abortion, premature rupture of membranes, and early-onset diseases (EOD) [33, 55]. It is worth noting that ST485 has become one of the leading STs in our region (accounting for 8.6%). Although all subtypes of CC61 and CC67 were previously reported as bovine strains [47, 53], ST929, which can cause threatened abortion in pregnant women, was first reported as a new subtype of CC67 in 2017 [33]. Furthermore, 2.8% of the colonized isolates in our study belong to ST929. The eBURST population snapshot (as of August 2018, number of STs = 1311) (see Figure 2) showed different CCs related to ST485 and ST929, which were previously grouped into CC103 and CC67, respectively [33]. This difference can be explained by the high genetic diversity observed and the dynamic nature of *S. agalactiae* in the MLST database [53].

One of the major objectives of this study was to investigate the gene-based antibiotic resistance of colonized *S. agalactiae*. In the current study, all strains were sensitive to penicillin, which was the first-line antibiotic for the treatment and prevention of *S. agalactiae* infection [10, 11]. Nevertheless, it is worth noting that our region has a high proportion of MDR strains. Erythromycin and clindamycin have long been considered as effective alternatives in IAP for penicillin-allergic women [9, 17]. However, the resistance rates for erythromycin and clindamycin in our region were 66.7% and 47.2%, respectively. Recently, such high resistance rates have also been observed in other Chinese cities, such as Beijing [16], Shanghai [29], and Dongguan [11]. Overall, the resistance rates for erythromycin and clindamycin in China were higher than those in other regions [2, 9, 18, 56]. Therefore, if erythromycin or clindamycin is used as an alternative antibiotic, antimicrobial susceptibility test should be performed in advance. Erythromycin resistance is usually associated with three macrolide resistance genes: *ermB*, *ermTR*, and *mefA/E* [17, 57]. Although the erythromycin resistance rates of *S. agalactiae* in different Chinese cities were similar, the resistance mechanisms were not the same. In this study, erythromycin resistance was mediated mainly by the *ermB* or *mef A/E* genes, and no isolates were found to carry the *ermTR* gene alone. The erythromycin-resistant strains carrying resistance genes in our study were different from those in previous reports [57]. The *linB* gene, encoding a lincosamide nucleotidyltransferase, confers moderate sensitivity to erythromycin and resistance to clindamycin [17, 18]. However, the *linB* gene was detected in two erythromycin-sensitive strains. This may be because the *linB* gene was not expressed, but further research is needed to confirm this inference.

In the current study, five main virulence genes of *S. agalactiae*, which encode the surface proteins that involved in adhesion, invasion, or immune evasion [58], were investigated. Similar to previous reports [27, 28, 49, 59], our report showed that the *cfb*, *scpB*, and *lmb* genes were distributed in most strains. However, the prevalence of the *bca* and *bac* genes in our study was different from that in previous reports. All strains in our study harbored the *bca* gene, indicating that the number of *bca*-positive strains was significantly higher in our region than that reported in other regions [29, 49, 59]. Our results indicated that the *bac* gene was identified only in serotype III (Table 2). This result was different from those reported around 2000, which showed that the *bac* gene was predominantly present in serotypes Ia, Ib, and II [27, 59]. These results suggested that the epidemiological distribution of *S. agalactiae* virulence genes might vary from period to period and region to region. Dmitriev A et al. used PFGE to show that the *bac*-positive strains were genetically homogeneous [59]. Instead, we found the that *bac* gene was distributed in different STs, suggesting that the *bac*-positive strains were genetically heterogeneous. The result of a previous study that used dot-blot hybridization was consistent with our inference [27]. This indicated that when PFGE was used as the major method for determining diversity within a population, the measure of diversity was missed [27]. We found that the *bca*-*cfb*-*scpB*-*lmb* profile was the most common gene cluster (Table 2), distributed in all serotypes and most CCs. Nevertheless, the correspondence analysis showed that the distribution of virulence gene clusters between the CCs and serotypes was different.

The ST17 strain, belonging to serotype III, was defined as “hypervirulent” *S. agalactiae*, associated with early onset meningitis and late-onset diseases (LOD) in neonates [39]. With the implementation of extensive screening and IAP strategies, the incidence of EOD has been greatly reduced [12, 39]. However, the morbidity rates of LOD remain unchanged [12, 39], partly because the ST17 strains are particularly capable of persisting in the vaginal flora [39]. Detection of “hypervirulent” ST17 strains in vaginal samples or in neonates should permit the identification of a neonate population that presents with high risk for *S. agalactiae* infection [60], and in such a population, antimicrobial therapy and strict follow-up are absolutely necessary [60]. In this study, ST17 was one of the most prevalent STs, and all ST17 strains belonged to serotype III. It is necessary to establish a rapid screening method for ST17 strains in pregnant women in this region. MALDI-TOF MS can rapidly distinguish among some bacterial subtypes using characteristic mass spectrum peaks [37]. Previous studies using MALDI-TOF MS (Bruker Daltonics) confirmed the characteristic mass spectrum peak of the ST17 strain at 7625 Da [2, 39]. However, we found this peak at 7620 Da using VITEK®MS. We speculated that the characteristic mass peaks of different detection systems are slightly different. ST106 was also found to harbor this peak [2], yet we did not identify this ST in this study. We hypothesized that MALDI-TOF MS is suitable for the rapid screening of the ST17 strains in our region.

With the continuous reduction in the cost of WGS and the rapid development of bioinformatic infrastructures [34], it is expected that WGS will be performed in the future to reveal more subtle characteristics of *S. agalactiae*. This study was limited in its small sample size, single-center design, and retrospective nature. Our next experiment will include an increased sample size. Overall, this study added new information on the antibiotic resistance and molecular characteristics of colonized *S. agalactiae* in China.

## 5. Conclusion

The data obtained in this study indicated that the main molecular epidemiological characteristics of colonized *S. agalactiae* in Guangzhou are similar to those in other regions. However, some regional characteristics are also shown, especially relatively high prevalence of highly pathogenic ST485 and ST929. Moreover, the high carrying rate of the *bca* gene and low carrying rate of the *bac* gene are also prominent features. This study facilitated the epidemiological comparison of different regions and the prevention of postpartum intrauterine and neonatal infections. Furthermore, due to the high multidrug resistance rate of the colonized strains in our region, anti-infective treatment based on antibiotic resistance monitoring is necessary. Finally, we hypothesized that MALDI-TOF MS is suitable for the rapid screening of the ST17 strains in Guangzhou.

## Figures and Tables

**Table 1:** Relationship between antibiotic nonsusceptibility, resistance genes, and serotypes.

Serotypes	Antibiotics^a^ (nonsusceptibility^b^)						Resistance genes (positive)			
	ERY	CLI	TET	LEV	CHL	MDR	*ermB*	*ermTR*	*mefA/E*	*linB*
Total (*n* = 72)	50 (69.4)	34 (47.2)	65 (90.3)	27 (37.5)	23 (31.9)	48 (66.7)	31 (62.0)	9 (18.0)	27 (54.0)	18 (52.9)
Ia (*n* = 13)	6 (46.2)	3 (23.1)	12 (92.3)	2 (15.4)	3 (23.1)	5 (38.5)	2 (33.3)	2 (33.3)	5 (83.3)	1 (33.3)
Ib (*n* = 16)	14 (87.5)	6 (37.5)	16 (100.0)	7 (43.8)	7 (43.8)	12 (75.0)	6 (42.9)	6 (42.9)	9 (64.3)	2 (33.3)
II (*n* = 3)	2 (66.7)	2 (66.7)	3 (100.0)	0 (0.0)	2 (66.7)	2 (66.7)	2 (100.0)	0 (0.0)	0 (0.0)	0 (0.0)
III (*n* = 38)	26 (65.8)	22 (57.9)	32 (84.2)	17 (44.7)	10 (26.3)	27 (71.1)	20 (76.9)	0 (0.0)	12 (46.2)	15 (68.2)
NT^c^ (*n* = 2)	2 (100.0)	1 (50.0)	2 (100.0)	1 (50.0)	1 (50.0)	2 (100.0)	1 (50.0)	1 (50.0)	1 (50.0)	0 (0.0)
*P* value^d^	0.148	0.159	0.483	0.194	0.333	0.183	0.054	0.001	0.213	0.093

*Note.* Values are *n* (%) or as otherwise indicated. ^a^All the strains were sensitive to penicillin, ampicillin, vancomycin, and linezolid. ERY, erythromycin; CLI, clindamycin; TET, tetracycline; LEV, levofloxacin; CHL, chloramphenicol; MDR, multidrug resistant. ^b^Nonsusceptibility includes intermediation and resistance. And no strains were found intermediate to clindamycin and levofloxacin in this study. ^c^Nontypeable. ^d^*P*-values was calculated with Fisher's exact test.

**Table 2:** Virulence gene profiles of 72 *S. agalactiae* isolates.

Virulence gene profiles	*n* (%)	Serotypes (number of isolates)	CC (number of isolates)
*bca*	1 (1.4%)	Ia (1)	CC485 (1)
*bca*-*cfb*	9 (12.5%)	Ia (5), Ib (1), and III (3)	CC61 (2), CC103 (1), and CC485 (6)
*bca*-*cfb*-*bac*	7 (9.7%)	III (7)	CC19 (1), CC103 (3), CC328 (1), and CC485 (2)
*bca*-*cfb*-*scpB*	1 (1.4%)	III (1)	CC103(1)
*bca*-*scpB*-*lmb*	5 (6.9%)	Ib (2), II (2), and NT (1)	CC8 (2), CC19 (2), and CC24 (1)
*bca*-*cfb*-*scpB*-*lmb*	44 (61.1%)	Ia (7), Ib (13), II (1), III (22), and NT^a^ (1)	CC8 (4), CC17 (8), CC19 (26), CC23 (2), CC24 (1), CC485 (2), and UT^b^ (1)
*bca*-*cfb*-*scpB*-*lmb*-*bac*	5 (6.9%)	III (5)	CC19 (4) and CC485 (1)

^a^Nontypeable. ^b^Untypeable.

**Table 3:** Association of antibiotic nonsusceptibility and resistance genes with sequence types.

ST (number of strains)	Erythromycin nonsusceptible		Macrolides resistance genes (positive)					Clindamycin resistance^a^	*linB* (positive)
	Intermediate	Resistant	*ermB*	*mefA/E*	*ermB + mefA/E*	*ermB + ermTR*	*mefA/E + ermTR*		
ST8 (1)	0 (0.0)	0 (0.0)						0 (0.0)	
ST24 (1)	0 (0.0)	0 (0.0)						0 (0.0)	
ST824 (1)	0 (0.0)	0 (0.0)						1 (100.0)	1 (100.0)
ST485 (6)	0 (0.0)	0 (0.0)						0 (0.0)	
ST27 (4)	1 (25.0)	3 (75.0)	2 (50.0)	2 (50.0)				0 (0.0)	
ST19 (22)	1 (4.5)	15 (68.2)	1 (6.3)	5 (31.2)	3 (18.8)	2 (12.5)	5 (31.3)	9 (40.1)	8 (88.9)
ST651 (5)	0 (0.0)	3 (60.0)	2 (66.7)		1 (33.3)			3 (60.0)	3 (100.0)
ST862 (6)	0 (0.0)	4 (66.7)	4 (100.0)					4 (66.7)	4 (100.0)
ST12 (5)	0 (0.0)	5 (100.0)	3 (60.0)		2 (40.0)			5 (100.0)	1 (20.0)
ST17 (8)	0 (0.0)	7 (87.5)	5 (71.4)		2 (28.6)			7 (87.5)	
ST885 (1)	0 (0.0)	1 (100.0)	1 (100.0)					1 (100.0)	
ST28 (2)	0 (0.0)	1 (50.0)	1 (100.0)					1 (50.0)	
ST86 (2)	0 (0.0)	2 (100.0)					2 (100.0)	0 (0.0)	
ST138 (1)	0 (0.0)	1 (100.0)		1 (100.0)				0 (0.0)	
ST328 (1)	0 (0.0)	1 (100.0)	1 (100.0)					1 (100.0)	1 (100.0)
UT^b^ (1)	0 (0.0)	1 (100.0)		1 (100.0)				1 (100.0)	
ST929 (2)	0 (0.0)	2 (100.0)		2 (100.0)				0 (0.0)	
ST23 (2)	0 (0.0)	1 (50.0)		1 (100.0)				0 (0.0)	
ST890 (1)	0 (0.0)	1 (100.0)	1 (100.0)					1 (100.0)	

*Note*. Values are *n* (%) or as otherwise indicated. ^a^No strains were found intermediate to clindamycin in this study. ^b^Untypeable.

## Data Availability

The data used to support the findings of this study are available from the corresponding author upon request.

## Supplementary Materials

Supplementary Materials Table S1: the MLST profiles of global dataset of *S. agalactiae*. These data include all *S. agalactiae* STs found worldwide as of August 2018 (XLS 57 kb). Table S2: antibiotic resistance combination of multidrug-resistant colonized *S. agalactiae* (docx 16 kb). Figure S1: characteristic mass spectrum analysis of different ST strains (PDF 271 kb).
