# Supplementary material for: Antibiotic Resistance and Molecular Epidemiological Characteristics of Streptococcus agalactiae Isolated from Pregnant Women in Guangzhou, South China
Source: Can J Infect Dis Med Microbiol. 2020 Apr 30;2020:1368942. doi: 10.1155/2020/1368942 (PMC7210523; doi:10.1155/2020/1368942)
Supplement: Supplementary Materials — Table S1: the MLST profiles of global dataset of S. agalactiae. These data include all S. agalactiae STs found worldwide as of August 2018 (XLS 57 kb). Table S2: antibiotic resistance combination of multidrug-resistant colonized S. agalactiae (docx 16 kb). Figure S1: characteristic mass spectrum analysis of different ST strains (PDF 271 kb). [file 1368942.f1.zip › 1368942.f1/Figure S1.pdf]

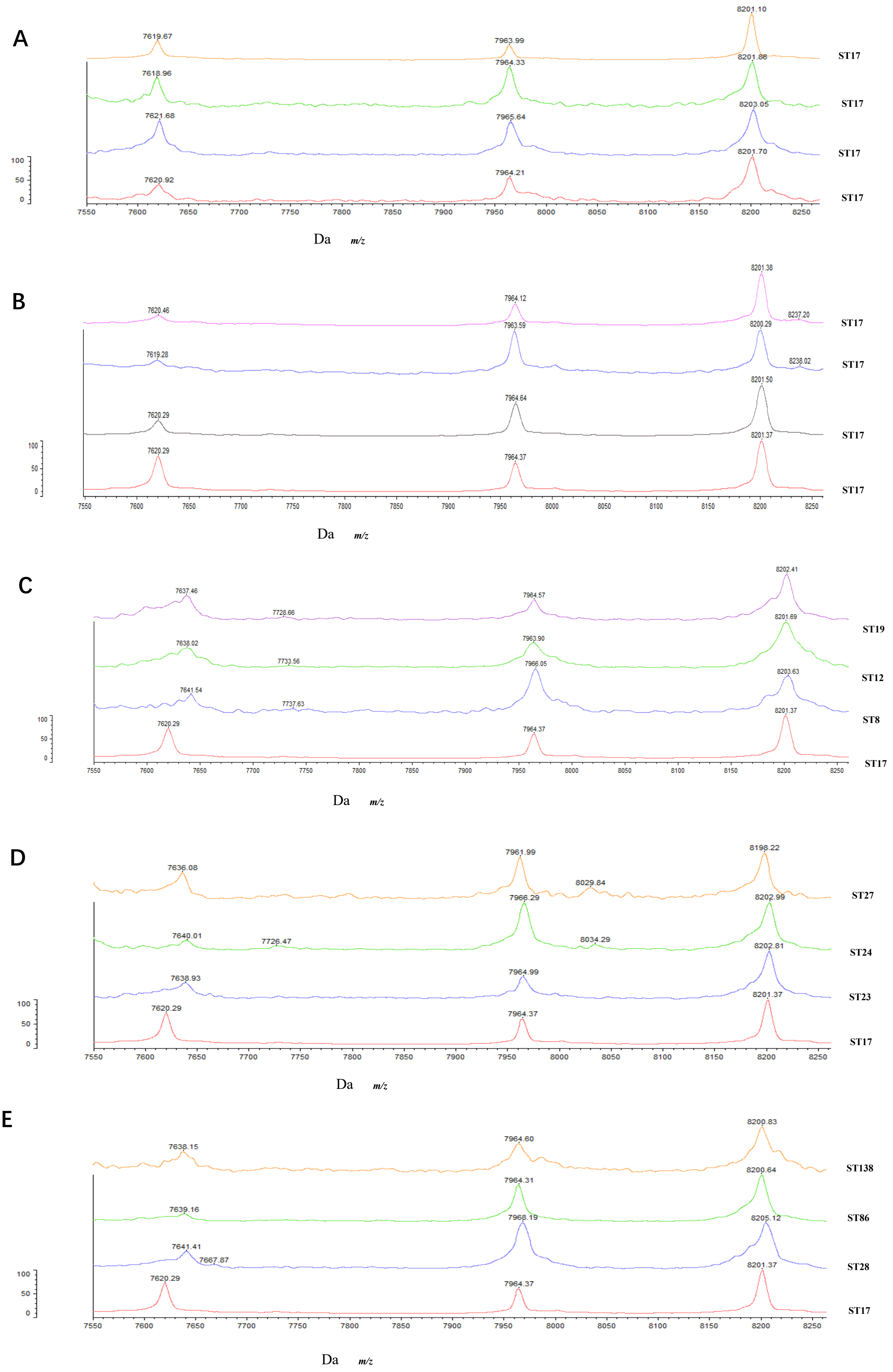
A
7619.67
7963.99
8201.10
ST17
7618.96
7964.33
8201.86
ST17
7621.68
7965.64
8203.05
ST17
7620.92
7964.21
8201.70
ST17
100
50
0
7550
7600
7650
7700
7750
7800
7850
7900
7950
8000
8050
8100
8150
8200
8250
Da m/z
B
7620.46
7964.12
8201.38
8237.20
ST17
7619.28
7963.59
8200.29
8238.02
ST17
7620.29
7964.64
8201.50
ST17
7620.29
7964.37
8201.37
ST17
Da m/z
C
7637.46
7728.66
7964.57
8202.41
ST19
7638.02
7733.56
7963.90
8201.69
ST12
7641.54
7737.63
7966.05
8203.63
ST8
7620.29
7964.37
8201.37
ST17
Da m/z
D
7636.08
7961.99
8029.84
8198.22
ST27
7640.01
7726.47
7966.29
8034.29
8202.99
ST24
7638.93
7964.99
8202.81
ST23
7620.29
7964.37
8201.37
ST17
Da m/z
E
7638.15
7964.60
8200.83
ST138
7639.16
7964.31
8200.64
ST86
7641.41
7667.87
7968.19
8205.12
ST28
7620.29
7964.37
8201.37
ST17
Da m/z

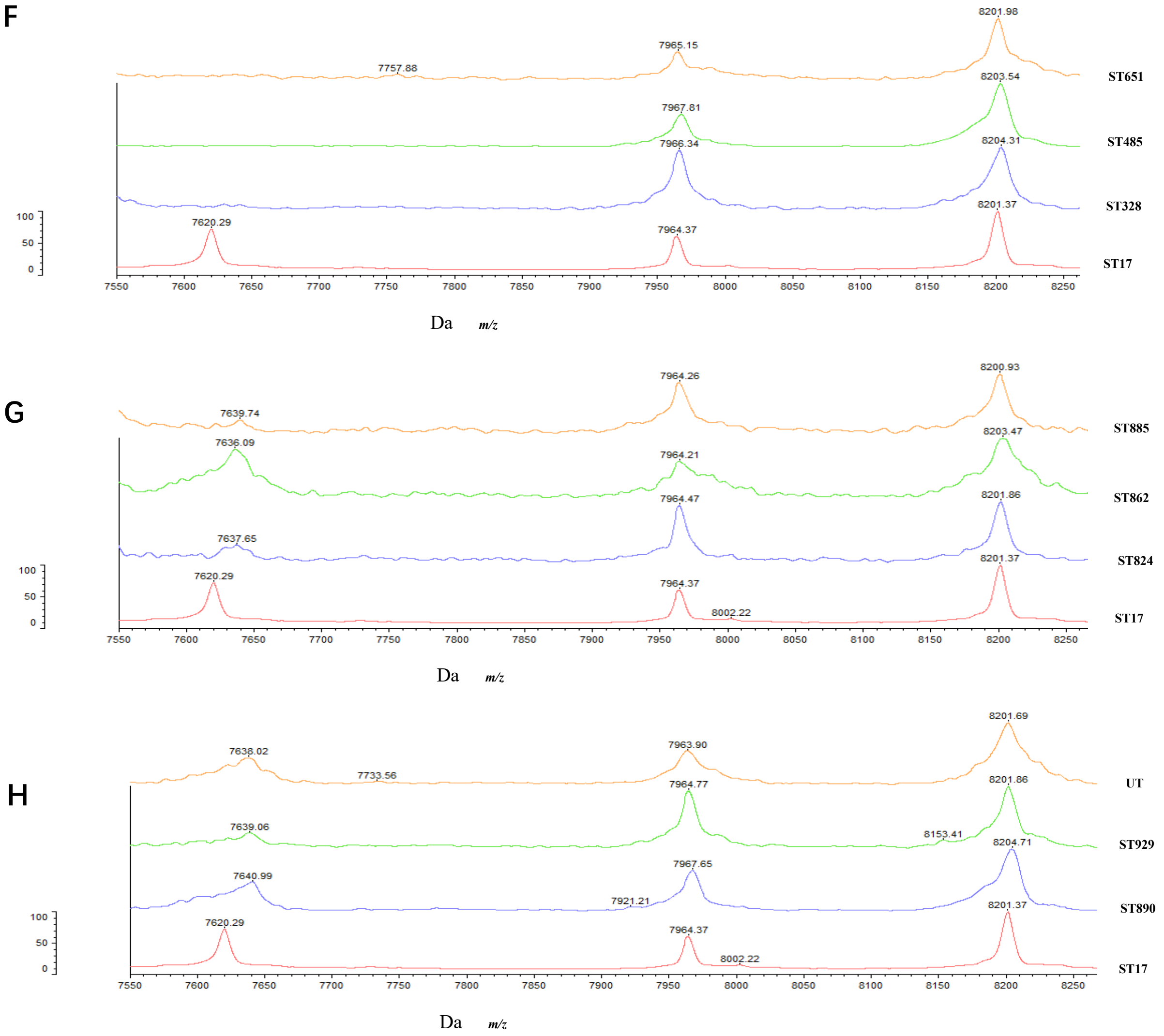

Figure S1. Characteristic mass spectrum analysis of different ST strains. Mass peaks of different ST strains were obtained by the Vitek MS system and analyzed with the research-use-only (RUO) SARAMIS premium software. The relative intensities of the ions are shown on the *y* axis, and the masses of the ions (in Da) are shown on the *x* axis. The m/z value represents mass to charge ratio. All eight ST17 strains have mass peaks at 7620 Da (A and B), distinguishing them from other STs strains in the range from 7550 to 8250 Da (C, D, E, F, G and H). UT represents the only untypable strain in this study.
