# Supplementary material for: Antibiotic Resistance and Molecular Epidemiological Characteristics of Streptococcus agalactiae Isolated from Pregnant Women in Guangzhou, South China
Source: Can J Infect Dis Med Microbiol. 2020 Apr 30;2020:1368942. doi: 10.1155/2020/1368942 (PMC7210523; doi:10.1155/2020/1368942)
Supplement: Supplementary Materials — Table S1: the MLST profiles of global dataset of S. agalactiae. These data include all S. agalactiae STs found worldwide as of August 2018 (XLS 57 kb). Table S2: antibiotic resistance combination of multidrug-resistant colonized S. agalactiae (docx 16 kb). Figure S1: characteristic mass spectrum analysis of different ST strains (PDF 271 kb). [file 1368942.f1.zip › 1368942.f1/Table S2.docx]

Table S2 Antibiotic resistance combination of multi-drug resistant colonized *S. agalactiae*.

| Class of antibiotic | Resistance combination | No. of isolates | Proportion of all isolates |
| --- | --- | --- | --- |
| 3 | erythromycin + clindamycin + tetracycline | 13 | 18.1% |
|  | erythromycin + clindamycin + levofloxacin | 1 | 1.4% |
|  | erythromycin + clindamycin + chloramphenicol | 2 | 2.8% |
|  | erythromycin + levofloxacin + tetracycline | 3 | 4.2% |
|  | erythromycin + tetracycline + chloramphenicol | 1 | 1.4% |
|  | clindamycin + levofloxacin + tetracycline | 6 | 8.3% |
| 4 | erythromycin + clindamycin + tetracycline + levofloxacin | 6 | 8.3% |
|  | erythromycin + clindamycin + tetracycline + chloramphenicol | 9 | 12.5% |
|  | erythromycin + levofloxacin + tetracycline + chloramphenicol | 7 | 9.7% |
